# Book Reviews

**Published:** 1923-06

**Authors:** 


### BOOK REVIEWS

NURSERY GUIDE FOR MOTHERS AND NURSES, by Louis W. Sauer, M. A., M. D. Senior attending pediatrician Evanston Hospital, formerly attending physician Chicago Infant Welfare and assistant attending physician Childrens' Memorial Hospital, Chicago. Published by the C. V. Mosby Company, 508 No. Grand Avenue, St. Louis, Missouri. Price, $1.75. Bound in cloth, illustrated and complete with necessary charts of statistics. 188 pages. This volume written for mothers and nurses, gives in a clear, concise form all essential details in the care and feeding of infants. Unlike other books, it does not give hazardous feeding formulas and prescriptions. It tells how to care for the infant in health and in disease. The subject-matter is coherent and systematically arranged. Dr. Louis W. Sauer, the author of this book, says that the care, nourishment and ills of infants are so different from those of children and adults that they deserve special consideration. The person in charge of an infant has unparelleled responsibility, for to her is entrusted the progeny of our race. Through providing proper care and nourishment, the ills of infancy are greatly reduced. Food, more than any of the other factors of selection, determines which child shall live. Babies fed food other than mother's milk as a rule are greatly handicapped in the struggle for existence.

Although epoch-making discoveries have been made in the science of infant feeding during the past two decades, a high infant mortality rate continues. Through the efforts of scientific investigators, physicians, departments of health, nurses and mothers, a decrease in the infant death rate is evident, but much still remains to be done.

This brief nursery guide is intended to aid those to whom are entrusted the care and feeding of infants.

This book can be obtained from the publishers, C. V. Mosby Co., St. Louis, Mo.

---

INTERPRETATION OF DENTAL AND MAXILLARY ROENTGENOGRAMS, by Robert H. Ivy, M. D., D. D. S., F. A. C. S. Professor of Maxillo-Facial Surgery, Graduate School of Medicine and of Clinical Maxillo-Facial Surgery School of Dentistry, University of Pennsylvania; Lieutenant-General Medical Reserve Corps, United States Army; and LeRoy M. Ennis, D. D. S., Instructor in Radiography, School of Dentistry, University of Pennsylvania; Captain Dental Reserve Corps, United States Army. Second edition revised and enlarged. Published by C. V. Mosby, 508 No. Grand Boulevard, St. Louis, Missouri. Price, $4.00. Bound in cloth and has 403 illustrations and 195 pages. As the title of this book indicates, it is an interpretation of Dental and Maxillary X-ray pictures. The purpose of this volume is to present to members of the medical and dental professions the data necessary for making an intelligent diagnosis of pathologic conditions about the teeth and jaw bones in which roentgen examination play a part. It is to interpretation rather than to technic that the writer has endeavored to call particular attention and to give especial emphasis, references to technique being limited to special points involved in examination of the teeth and jaw bones. This book is very complete and comprehensive, covering all kinds of cases that the hospitals, especially the clinics of the Thomas W. Evans Institute, have experienced. The practical features of this book are of immense value to the reader. This volume can be obtained from the publishers, C. V. Mosby Co., St. Louis, Mo.

